# Nuancing the role of social skills– a longitudinal study of early maternal psychological distress and adolescent depressive symptoms

**DOI:** 10.1186/s12887-018-1100-4

**Published:** 2018-04-10

**Authors:** Wendy Nilsen, Evalill Bølstad Karevold, Jannike Kaasbøll, Anne Kjeldsen

**Affiliations:** 1 Work Research Institute, OsloMet – Oslo Metropolitan University, Postbox 4, St. Olavs plass, 0130 Oslo, Norway; 2 Department of Mental Disorders, Norwegian Institute of Public Health, Postbox 4404, Nydalen, 0403 Oslo, Norway; 3 Department of Psychology, University of Oslo, Oslo, Norway; 4 Department of Health Research, SINTEF Technology and Society, Klæbuveien 153, Trondheim, 7049 Norway; 5 Department of Child Development, Norwegian Institute of Public Health, Postbox 4404, Nydalen, Oslo, 0403 Norway; 6 Bjørknes University College, Lovisenberggata 13, 0456 Oslo, Norway

**Keywords:** Social skills, Adolescence, Depression, Maternal depression, Longitudinal studies

## Abstract

**Background:**

Social skills might play an important role for the relationship between maternal psychological distress and subsequent development of depressive symptoms. The majority perspective is that social skills is adaptive and protective, but there is a need to also highlight the potential maladaptive effect of social skills in some settings or for some sub groups.

The current study examined the longitudinal interplay between maternal-reported psychological distress in early childhood (age 1.5), and offspring reports on social skills and depressive symptoms in early (age 12.5) and middle adolescence (age 14.5).

**Methods:**

We used data from the Tracking Opportunities and Problems Study (TOPP), a community-based longitudinal study following Norwegian families to examine direct links and interactions between early maternal distress (measured with the Hopkins Symptom Checklist) and early adolescent offspring social skills (measured with the Social Skills Rating System) and middle adolescent depressive symptoms (measured with the Moods and Feelings Questionnaire) in 370 families (in total 740 mothers and adolescents).

**Results:**

Exposure to childhood maternal distress predicted offspring depressive symptoms in middle adolescence. Higher social skills in early adolescence predicted lower levels of depressive symptoms for girls, but not for boys, in middle adolescence. An interaction effect was found in which adolescents exposed to early maternal distress who reported high social skills in early adolescence had the highest level of depressive symptoms in middle adolescence.

**Conclusions:**

The findings highlight the nuances in the role of social skills for adolescent depressive symptoms – having the potential to be both adaptive as well as maladaptive for some subgroups (those experiencing maternal psychological distress). This has important implications for social skill programs.

## Background

Adolescence is a well-documented period for the development of depressive symptoms [1–3]. Adolescent sub-threshold levels of symptoms are associated with diagnosed depression and other adverse outcomes in adulthood [4, 5]. Studying factors and mechanisms that contributes to early identification of depressive symptoms *before* they become chronic is therefore essential for effective intervention and prevention. One single factor alone (e.g., gender, maternal distress, temperament) is seldom responsible for the development of internalizing problems. In line with the biopsychosocial model of development and developmental psychopathology, an integrative perspective including both contextual and individual factors is likely to extend our understanding of the complex etiology of depressive symptomatology. Maternal psychological distress (i.e., symptoms of anxiety and depression) is one the most well-known early familial/contextual risk factors for depressive development in adolescents [6–8], and social skills are shown to be a protective individual factor in adolescence [9–13]. However, the impact of the interplay between early maternal distress and social skills on the development of depressive symptoms in adolescence is less examined. Thus, in the current paper, we aim to study the interplay between early childhood maternal psychological distress (age 1.5) and early adolescent social skills (age 12.5) to predict depressive symptoms in middle adolescence (age 14.5).

### Maternal psychological distress and adolescent depressive symptoms

There is an increased risk of psychological distress in offspring when parents experience psychological distress [6–8], even for mild levels of depressive symptoms [14–16]. The transitional mechanisms are suggested to work through both biological (genetic or in utero transitions) [17, 18] and environmental influences (i.e., less sensitive, and emotional unavailable parenting) [8, 19], or through a combination of these [20]. The framework of developmental psychopathology highlights the possibility that similar types of stressors, such as maternal depression, may have different effects at different developmental stages [21, 22]. Theories of early life vulnerability for instance, emphasize the possibility of sensitive stages where stressors may have a particular strong impact early in life as compared to other stages [23]. In line with this, early exposure to maternal psychological distress is suggested to interfere with the development of important processes and competencies in childhood, such as attachment, emotion regulation, interpersonal skills, and stress responses, which can give a heightened risk for subsequent development of depression [24]. Albeit several studies support the long-term adverse impact of early sensitive period of experiencing maternal depressive symptomatology [8, 25], there is less research examining the potential impact social skills might have on the association between maternal psychological distress and the development of subsequent adolescent depressive symptomatology. A recent review emphasized the need for studies identifying processes accounting for positive outcomes such as social skills for children and adolescents with depressed mothers [26].

### Maternal psychological distress, social skills and adolescent depressive symptoms

Good social skills is defined as being able to interact with other people in a way that is both appropriate (e.g., not eliciting negative responses from others) and effective (e.g., achieving one’s goal with the interaction) [13]. The social skills construct contains different dimensions, like perspective taking (cognitive), empathy (emotional), and cooperation and self-control (behavioral) [27]. Despite social competence being defined as a broader term that includes social skills as well as other behavioral, cognitive, and emotional traits necessary to develop and maintain adequate social relationships, the terms social skills and social competence are often used interchangeably [28]. Thus, in the current paper we will refer to studies focusing on both social competence and skills. Good social skills can make it easier to manage stressors and challenges connected to developmental transitions such as adolescence, and thus protect the development of subsequent adverse outcomes. Supporting this, cross-sectional and longitudinal findings report good social skills to be associated with less depressive symptoms in adolescence [9–13].

Based on the social skills deficit theory [29, 30], the social skills deficit vulnerability model argues that low social skills create vulnerability for developing psychosocial problems when experiencing stress [13, 31]. While some studies examining exposure to stressful events support this hypothesis [13, 31, 32], few have examined exposure to maternal depression or psychological distress as example of experiencing stress. Still, studies examining terms such as social functioning (i.e. functioning in social life, close friendship, romantic and family relationship), which is related to social skills, have examined the interplay between maternal depression and depression in their offspring [33–35]. A recent study reported that high social functioning was an important protective factor for adolescent offspring of depressed parents, with a twofold increase of having good mental health for those displaying high social functioning [35]. Also, another study reported an interaction effect in which depressive symptoms was higher for adolescent offspring of depressed mothers with low interpersonal functioning in a cross-sectional study [33]. This was supported in a longitudinal five-year follow-up study of the same sample [33, 34]. With the strength of conducting clinical interviews of both mothers and children, these studies indicate a complex interplay between interpersonal factors, maternal depression and adolescent depression. The current study aimed to expand upon these studies to examine social skills capturing abilities such as empathy, cooperation, assertiveness and self-control in adolescents.

While the social skills deficit theory offers a valuable insight in the protective trait of social skills or the negative effect of social skills deficits, a more nuanced theoretical perspective advocates a view of social skills as both adaptive *and* maladaptive. Despite its initial counter-intuitiveness, researchers have suggested that social skills can be used in maladaptive manners, in that individuals might take into consideration others so much that it overrule their own needs [36]. Additionally, having higher social skills can be used as a means to manipulate others behavior, which have been indicated by findings linking bullying behavior and high social skills in some groups of adolescents [37]. Researchers have indicated that being emphatic, a prosocial trait, as a response to parental depressive symptoms might be both adaptive and maladaptive [38]. Findings from a recent study support this notion [39]. This finding showed that high empathy was associated with higher internalizing problems in children with depressed mothers, but not in children with non-depressed mothers, indicating that social skills are not always protective. It was hypothesized that high level of empathy can lead to emotional overarousal when experiencing others’ pain or discomfort. A nuanced perspective of social skills is thus important, because of the changeable nature of these skills, which is a main element of many prevention/ intervention programs [40, 41]. In a worst-case scenario, social skills training programs that do not inhabit this nuancing perspective might increase vulnerability to contextual stressors for some subgroups of children and adolescents. No published studies have, to our knowledge, examined whether social skills during early adolescence are adaptive or maladaptive in the context of exposure to childhood maternal psychological distress with comprehensive longitudinal multi-informant design.

### Gender differences

It is important to gain more knowledge on gender-specific pathways to the development of depressive symptomatology in adolescence, due to the consistently reported finding that girls report higher depression levels from puberty and onwards [2, 3, 42]. Girls are two to three times more likely to report depressive symptoms in both population-based and clinical samples than boys are [2, 3, 42–44]. Gender-specific vulnerability to maternal psychological distress seems more inconsistent [45]. Findings indicate an interaction between gender and developmental stage in which boys seems more vulnerable for maternal distress in infancy (e.g., [46]), while girls seem to more vulnerable during adolescence [45, 47]. However, there are also studies showing no gender differences [48, 49].

It is suggested that girls, compared to boys, are especially reactive to interpersonal stressors due to increased impact of hormones and gender socialization [42, 50]. Findings supporting this is however contradictory with some studies reporting girls to be more interpersonally vulnerable compared to boys (i.e., stronger associations between social skills and depressive symptoms) [50], while other studies are inconclusive with no gender differences [12]. Similarly, adolescent girls report higher social skills in some studies [51], and lower social skills in other studies compared to boys [52–54]. In the few studies examining the interplay between maternal depressive symptomatology and offspring social skills and depressive symptoms, some gender-specific findings have emerged. A study reported that social skills mediated the association between maternal depression and adulthood depression in female, but not male offspring [55], indicating a specific interpersonal stressor-related pathway to depressive symptoms for adult female offspring having experienced maternal depression.

### The current study

In accordance with former findings [33–35], we predict that: 1) Higher levels of maternal psychological distress at age 1.5 predict higher depressive symptoms at age 14.5, and 2) Higher social skills at age 12.5 predict lower depressive symptoms at age 14.5. Due to few former findings and in light of the nuancing view of social skills, we examine the following exploratory research questions: 3) Do social skills at age 12.5 positively or negatively affect the relation between maternal psychological distress at age 1.5 and depressive symptoms at age 14.5 (i.e., interaction effects)? and 4) Are there gender differences in the interplay between early maternal psychological distress, social skills and depressive symptoms?

## Methods

### Participants and procedure

Families from 19 geographic health care areas in eastern Norway were invited to complete a survey questionnaire when attending the 18-month vaccination visit for the index child in 1993 (Time 1 (T1)) [56, 57]. Regional Committees for Medical and Health Research Ethics (REC) approved the data collection. The 19 health care areas differed considerably and were overall representative of the diversity of social environments in Norway^34^. Routinely, more than 95% of all Norwegian families with children attend a public health program during the first 4 years of the child’s life. Of the 1081 invited families, 929 families (86%) participated at T1. Families participating at T1 received a similar questionnaire when the children were ages 2.5 (T2), 4.5 (T3), 8.5 (T4), 12.5 (T5), 14.5 (T6), 16.5 (T7) and 19 (T8). Mothers completed questionnaires at all waves, fathers were included from T6, and the children/adolescents themselves completed questionnaires from T5.

Since the main interests were examining maternal distress in early childhood, and early adolescent social skills and depression, we used reports at ages 1.5, 12.5 and 14.5 for the current study. Of the 913 mothers who participated at T1, 481 (52% of the T1 sample) also participated at T6, 566 children (61% of the T1 sample of mothers) participated at T5 and 458 (50%) at T6 with an even gender distribution (51% girls).

The current sample included 370 mothers-child dyads (*n* = 740) with mothers reports on T1 and T6, and adolescents’ report on T5 and T6. Adolescents responded to social skills for the first time at age 12.5 (T5). Eight individuals were excluded from the analyses, due to more than half of the information missing on one of the main variables of interests, leaving the sample to be 362 mothers-child dyads, with 159 mothers-son dyads (*n* = 308), and 203 mother-daughter dyads (*n* = 406).

Baseline background information showed that non-participating mothers did not significantly differ from participating mothers in maternal age, education, employment status, number of children and marital status [56]. Attrition analyses indicated that of several variables (e.g., maternal education, maternal work participation, family financial stress, marital status, stressful life events and depressive symptoms), only low maternal educational level predicted dropout for mothers [58, 59] and adolescents [60, 61]. Male gender predicted dropout for adolescents [60, 61]. Associations between mental health and the other examined variables at baseline (e.g., maternal education, maternal work participation) did not differ among dropout versus remaining families, suggesting that estimated associations between variables are generalizable [58].

### Measures

Maternal psychological distress was assessed with the 25-item version of the Hopkins Symptoms Check List (HSCL-25) [62–64], which has shown good psychometric qualities both internationally and in Norwegian samples for the measurement of symptoms of depression and anxiety [62–65]. The responses are rated on a four-point scale ranging from 1 “Not at all” to 4 “Extremely” affected. The items “thought of ending my life” and “loss of sexual interest or pleasure” were excluded from the questionnaire in T1 and T6 because they were perceived as offensive in a pilot study. A total mean score was calculated based on these 23 items, with a Cronbach alpha (internal reliability) of .90 at both waves in the current study.

Adolescent depressive symptoms were assessed with the Short Mood and Feelings Questionnaire (SMFQ) [66, 67]. The SMFQ consists of 13 questions drawn from original 34-item Moods and Feelings Questionnaire, with the response categories “Seldom true”, “Sometimes true” and “Often true”. One item, “I had difficulties concentrating” was not included in the questionnaire because of content overlap. One item (‘I feel lonely’) was similar to some constructs from the SSRS scale (see below). Analyses excluding this item did not change the results, so the item was included in all analysis. The SMFQ has shown satisfactory psychometric qualities in both international [66, 67] and Norwegian samples [68, 69]. A total mean score was used, with a Cronbach alpha (internal reliability) of .88 in the current study.

Social skills were assessed with a shortened form of the Social Skills Rating System (SSRS) at T5 [70]. The SSRS has shown satisfactory psychometric qualities in both international [70, 71] and Norwegian samples [72–74]. Reliability and factor analyses of the original 39 item scale at former time points in the TOPP-study (T4) assessed with parental reports, led to the development of a 24-item version used for subsequent waves (unpublished findings). Former publications imply that the 24-item version has predictive validity [75]. The Cronbach alpha (internal reliability) for the shortened form of SSRS was high (.88) for the current sample. SSRS measures four social skills dimensions: Cooperation (six items), Assertiveness (six items), Empathy (six items) and Self-control (six items). The response categories were originally three (“Never”, “Sometimes”, and “Very Often”), but two extra response categories (“Seldom”, and “Often”) was added in the Norwegian version after former recommendations [72]. An index was constructed by computing the mean score for the total scale.

Possible confounder variables included maternal psychological distress at T6 and socio-economic indicators (SES) at T1 composed of maternal education, maternal work participation, and family financial stress.

### Analytic strategy

Statistical analysis was performed using SPSS (Statistical Package for the Social Sciences). T-tests were conducted to examine gender differences in adolescent depressive symptoms, social skills and maternal psychological distress. Hierarchical block regression analysis was used to examine direct effects and interaction effects on self-reported adolescent depressive symptoms at T6 on adolescent girls and boys separately Table 1.

**Table 1:** Characteristics of participating families at baseline (T1)

Variables	
Maternal age (years), mean (SD)	30 (4.7)
Maternal education %	
Basic schooling (≤9 years)	9.5%
Basic schooling+ (10–11 years)	27.6%
Finished high school (13 years)	25.4%
Higher education (≥14 years)	37.6%
Maternal work status %	
No paid work	37.0%
Part-time paid work	32.0%
Full-time paid work	31.0%
Maternal relationship status %	
Married/cohabiter	91.2%
Single	8.8%
Maternal mother tongue %	
Norwegian	93.6%
Other than Norwegian	6.4%
Family economy %	
We manage well/very well	53.4%
We manage	40.7%
We manage poorly/very poorly	5.9%
Pariety %	
One child	48.2%
Two children	37.2%
Three to ten children	14.6%
Offspring infant gender %	
Girl	51.1%
Boy	48.9%

The mean score of adolescent depressive symptoms was log transformed to compensate for skewed data. In the first step, maternal distress at T1 and self-reported adolescent social skills at T5 were inserted to examine direct effects. Following, a variable constructed by the product between the standardized value of maternal distress at T1 and the standardized value of self-reported adolescent social skills at T5 was inserted representing the interaction effect. In the final step, we adjusted for SES at T1 and concurrent maternal psychological distress at T6.

## Results

See 1 for descriptive characteristics of the sample at baseline. The age of the mothers ranged from age 19 to 46 (M = 30; SD = 4.7), and a minority of the mothers (9%) were single. Eight percent of the mothers had 9 years schooling or less, while 18% had a college or university education of 4 years or more. Roughly, equal numbers of mothers worked fulltime (32%), part-time (31%), or had no paid work (37%).

Table 2 presents the descriptive information of all variables and t-tests of gender differences. Adolescent girls scored significantly higher than boys did on depressive symptoms and social skills with medium effect sizes. There were no gender differences in experiences of early maternal psychological distress. Tables 3 and 4 show correlations for adolescent girls and boys, respectively. While the associations between early and concurrent maternal distress and adolescent depression were significant for both girls and boys, associations between early adolescent social skills and adolescent depressive symptoms were only significant for adolescent girls. Early maternal distress did not correlate with adolescent social skills for either girls or boys.

**Table 2:** Descriptive information and t-tests of gender differences in the main variables

Variables	Males (*n* = 162–166^+^)		Females (*n* = 203–204^+^)		Total (*n* = 365–370^+^)				
	Mean	(SD)	Mean	(SD)	Mean	(SD)	t		d
Early maternal distress (age 1.5)	1.30	(.28)	1.32	(.31)	1.31	(.29)	−.56		.07
Social skills (age 12.5)	4.04	(.45)	4.19	(.42)	4.12	(.44)	−3.21	**	.34
Depressive symptoms (age 14.5)	.23	(.28)	.43	(.40)	.34	(.36)	−5.64	***	.58

* *p* < .05, ** *p* < .01, *** *p* < .001

^+^Samples sizes vary due to missing information at some waves

**Table 3:** Pearson’s correlations between the main variables in adolescent girls (*n* = 203)

Variables	1	2		3		4		5	
1. Adolescent depression (age 14.5)	–	.19	**	−.30	**	.16	**	−.10	
2. Maternal distress (age 1.5)		–		−.05		.36	**	−.32	**
3. Social skills (age 12.5)				–		−.15	*	.08	
4. Maternal distress (age 14.5)						–		−.20	**
5. Sosioeconomic indicators								–	

*p < .05; **p < .01

**Table 4:** Pearson’s correlations between the main variables in adolescent boys (*n* = 159)

Variables	1	2		3		4		5	
1. Adolescent depression (age 14.5)	–	.16	*	.00		.16	**	.00	**
2. Maternal distress (age 1.5)		–		−.10		.34	**	−.25	**
3. Social skills (age 12.5)				–		.04		−.06	
4. Maternal distress (age 14.5)						–		−.09	
5. Sosioeconomic indicators								–	

*p < .05; **p < .01

Tables 5 and 6 present findings from hierarchical block regression analysis for boys and girls separately. In step 1, early maternal distress significantly predicted adolescent depressive symptoms for both girls and boys. While social skills in step 2 were a significant negative predictor and increased the explained variance of adolescent girls’ depressive symptoms, it was not a significant predictor for boys.

**Table 5:** Maternal distress and adolescent social skills as predictors of depressive symptoms in adolescent girls (*n* = 203)

Predictors	Model 1			Model 2			Model 3			Model 4		
	*ß*	*t*		*ß*	*t*		*ß*	*t*		*ß*	*t*	
Early maternal distress (age 1.5)	.21	3.10	**	.23	3.41	***	.19	2.60	**	.16	1.96	
Adolescent social skills (age 12.5)				−.29	−4.37	***	−.28	−4.09	***	−.27	−3.87	***
Maternal distress x social skills							.08	1.01		.08	1.02	
Current maternal distress(age 14.5)										.06	.81	
Socioeconomic indicators										−.02	−.31	
*R*		.045			.129			.133			.137	
*R* ^*2*^_*adj*_		.041			.120			.120			.115	
*F*_*change*_		9.58	**		19.07	***		1.02			.40	

* *p* < .05; ** *p* < .01; *** *p* < .001

**Table 6:** Maternal distress and adolescent social skills as predictors of depressive symptoms in adolescent boys (*n* = 159)

Predictors	Model 1			Model 2			Model 3			Model 4		
	*ß*	*t*		*ß*	*t*		*ß*	*t*		*ß*	*t*	
Early maternal distress (age 1.5)	.21	2.67	**	.21	2.68	**	.25	3.13	**	.21	2.43	**
Adolescent social skills (age 12.5)				.02	.30		.01	.17		.00	.01	
Maternal distress x social skills							.19	2.40	**	.19	2.37	**
Current maternal distress (age 14.5)										.09	1.01	
Socioeconomic indicators										.00	.03	
*R*		.044			.044			.078			.084	
*R* ^*2*^_*Adj*_		.037			.032			.060			.054	
*F*_*change*_		7.15	**		.07			5.77	*		.51	

* *p* < .05; ** *p* < .01; *** *p* < .001

The interaction term of early maternal distress and early adolescent social skills added in step 3 was a significant positive predictor and increased the explained variance significantly for adolescent boys’ depressive symptoms, but not for girls (see F_change_ scores in Tables 5 and 6).

When adjusting for concurrent maternal distress and socio-economic indicators, the majority of the effects in step 3 remained for both girls and boys. Only the effect of early maternal distress on adolescent girls’ depressive symptoms became nonsignificant (*p* = .052). Before conducting adjustments, the final model explained more variance in adolescent girls’ (12%) compared to boys’ depressive symptoms (6%).

**Fig. 1:**
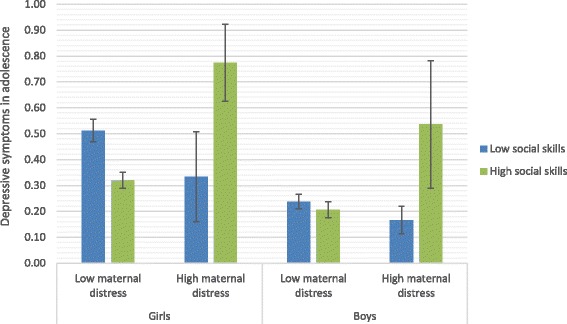
Mean levels of self-reported symptoms of depression in adolescent boys and girls (age 14.5) (with confidence intervals based on standard errors) for high versus low maternal distress and social skills

Post hoc graphical figures were conducted to explore the direction of the interaction. To do so, we dichotomized the measure of maternal distress according to former recommendations of a cut-off score of mean = 1.75 [76, 77], and conducted median-split of social skills (due to lack of cut-off recommendations the short form version of 24 items). See Fig. 1 for a graphical depiction of the interaction effects. Confidence intervals show that for those experiencing no or low maternal psychological distress in childhood, high social skills is associated with lesser depressive symptoms, than low social skills. This was found or girls only. For those experiencing early maternal distress, this pattern was vice versa. For both boys and girls experiencing early maternal distress and reporting high social skills in adolescence the level of depressive symptoms was significantly higher (as indicated by no overlapping confidence intervals) than all other groups. The three other groups had overlapping confidence intervals showing no significance difference between these groups. The wider confidence intervals for the high maternal distress groups are due to smaller sample sizes in both the groups of girls and boys having experienced high maternal distress.

## Discussion

The current study examined the interplay between early childhood maternal psychological distress and early adolescence social skills and adolescent depressive symptoms within the perspective of early life vulnerability theories. In line with our predictions, we found a prospective 12-year longitudinal link between early maternal distress and depressive symptoms in middle adolescence (age 14.5), supporting former studies showing that early childhood is a sensitive period for the exposure to maternal distress [6–8, 59]. This pathway was present for both girls and boys, and persisted when adjusting for confounding variables for boys, but became borderline significant for girls. Despite not being a surprising finding, the link between early childhood maternal distress and adolescent depressive symptoms is a prerequisite for our later predictions.

Our second prediction of a direct pathway from early adolescence social skills (age 12.5) to middle adolescence depressive symptoms (age 14.5), with low levels of social skills predicting higher depressive symptoms, was also supported. However, this pathway was only evident for girls, supporting theories and findings of girls being more interpersonally vulnerable compared to boys [42, 50, 78]. There might be other interpersonal factors not captured by the SSRS that might be relevant for boys, as suggested by a former study using the same data material as the current study [59]. In this former study, boys’ development of internalizing symptoms were significantly more affected by high levels of shyness and low activity levels compared to girls [59]. The current finding underscores the gender-specific direct pathway further from total social skills to depression development for girls.

Third, we found that for children who were exposed to maternal distress in early childhood, higher social skills in early adolescence increased the level of depressive symptoms in middle adolescence. These findings are in contrast to former theories and findings of social skills generally having a stress-buffering protective effect [6, 13, 31, 32, 79, 80], but support recent indications of social skills being maladaptive in certain settings [36, 38, 39]. This is also in contrast to former findings reporting high social functioning behavior to be protective [33–35]. When measuring maternal distress and social skills as continuous variables, this was only found for boys. However, when examining dichotomous variables (i.e., high versus low distress and social skills) this interaction was found for both girls and boys. In line with theories suggesting social skills to be protective, we also found that for adolescent girls exposed to *no or low* maternal distress in early childhood, higher social skills were protective against depressive symptoms.

Our findings highlight the importance of having a nuanced picture of social skills. While the social skills deficit theory offer a valuable insight in the adverse effects of social skills deficits, there is need for a more nuanced perspective also incorporating the possibility of high social skills to be maladaptive in some settings. Despite the counter-intuitiveness of the notion that social skills might be maladaptive for certain sub groups (i.e., children experiencing early childhood stress such as maternal distress), they are in line with both former suggestions that social skills can make individuals to be too considerate towards others, forgetting their own needs, or can be used as a means to manipulate others [36–38]. Moreover, a recent study reported that being emphatic as a response to parental depressive symptoms might be maladaptive [39]. The reason for few other empirical findings of the potential maladaptive effect of social skills, might be due to difficulties in publishing finding that counter the established understanding of social skills as adaptive. It is essential to replicate these findings in other samples using a hypothesis-testing approach for the validation and generalization of the findings to other samples. For instance, we did not examine the potential effect of early paternal psychological distress, as the fathers of the adolescents did not participate in the first five waves. Considering the increasing number of fathers participating in their children’s life today, and emerging findings on the link between paternal and offspring psychopathology [81, 82], future studies are encouraged to also include fathers. Moreover, future studies should also aim to examine the mechanisms underlying this phenomenon, such as emotional over arousal when experiencing other’s pain or discomfort.

Despite being outside the focus of the current study, the correlation analyses revealed an interesting lack of association between early maternal distress and subsequent adolescent social skills for both girls and boys. This is in contrast to some former findings (e.g., 45, 55). Potential reasons for this is examining a community-based sample (as opposed to a clinical sample) or the longer follow-up period as compared to former studies. Moreover, it might be that the current sample lacks the most severe cases of maternal psychological distress that might affect long-term social skills [See also limitation section for thorough discussion on dropout rates and generalizations].

### Strengths and limitations

The current study benefited from a 13-year longitudinal prospective community sample of 362 mother-child dyads (*n* = 724 individuals), with separate informants for maternal psychological distress (i.e., mothers’ self-reports) and adolescent social skills and depressive symptoms (i.e., adolescents’ self-reports), as well as well-validated psychometric instruments. There are still several limitations. First, the use of self-report and not observational data increases the risk for shared method variance to inflate the strength of associations. However, the use of multiple waves and two informants (i.e., mothers’ reports on their psychological distress, and adolescent’s reports on their social skills and depressive symptoms) reduces this common method bias. Moreover, using adolescent’s own reports instead of maternal reports also reduces potential depression-distortion bias (i.e., that mothers who score high on depressive symptoms might report their children’s symptoms more negatively).

Second, we did not examine reciprocal effects between depressive symptoms and social skills across childhood in children and their mothers because the children were only invited to participate in the study with self-reports from adolescence and onwards. Parallel symptom development in family member might however affect each other, as reported in other studies and should be pursued in future studies [83].

Third, the instrument used to measure social skills, SSRS has been updated after our study was conducted, to “the Social Skills Improvement System-Rating Scales” (SSIS-RS) [84]. The changes have led to inclusion of more subscales (e.g., “communication”), and studies in both Norway and North America shows that the new instrument had significantly higher reliability scores on some of the overlapping subscales [74, 84]. However, the old instrument (SSRS) was found to correlate highly with the new instrument (SSIS-RS) [74]. Future studies should consider using the new instrument.

Also, respecting pilot study participants’ wishes we deleted two items from the Hopkins Symptom Check List (i.e., “thought of ending my life” and “loss of sexual interest or pleasure”) – thus leaving out some variation.

Finally, as expected in a longitudinal prospective study - the dropout rate in the current sample proposes caution in generalizing the findings to the other populations. Attrition rates as high as 40–60% is not uncommon in longitudinal studies and only systematic non-random drop-outs represent a problem [84]. The only systematic difference between drop-outs and remaining participants was low level of education and male gender [58–61]. However, a range of other indicators such as work participation, family financial stress, marital status, stressful life events and depressive symptoms did not affect dropping-out, and associations between variables at baseline did not differ among drop-out versus remaining families later in the TOPP-study [58], suggesting that estimated associations between variables are generalizable. Thus, we believe that our sample is representative of similar samples of “normal”, but not to the same extent representative of high risk populations. Note however that the Norway is characterized by less inequality due to a redistribution of resources by the welfare state [85]. This suggests that despite drop-out being predicted by low educational status, the sample might still be generalizable to the majority of Norwegian families. As such, the findings might be more easily generalized to other Northern European societies that are similar to Norway with regards to the social welfare system as opposed to societies that are very different. Still, due to the risk of associations being underestimated in samples including parents with higher education, we suggest that future studies to be persistent in their recruitment of families from all layers of society.

## Conclusions

The current study examined the longitudinal interplay between early maternal distress (age 1.5) and adolescent offspring social skills (age 12.5) and depressive symptoms (age 14.5) in 362 mothers-adolescents dyads. The findings highlight the importance of examining the complex interplay between both individual (e.g., social skills) and familial/contextual variables (e.g., maternal distress) in their prediction of adolescent internalizing problems. The findings replicate the established link between childhood maternal distress and adolescent depressive symptoms, but also shed light on the role of social skills for this link. The results of the current study indicate that high social skills in early adolescence can be adaptive, in its direct link to subsequent low depressive symptoms 2 years later. The most noteworthy findings is however that high social skills for some individuals (i.e., those with mothers reporting high psychological distress) is maladaptive (i.e., linked to higher depressive symptoms in adolescence). It is thus important that social skills training efforts are able to tailor their programs to both those needing help to increase their use of social skills trait, but also to those needing help to put their own needs in front of others in some settings. More research is needed to further examine these nuances in social skills.
